# Can the vector space model be used to identify biological entity activities?

**DOI:** 10.1186/1471-2164-12-S4-S1

**Published:** 2011-12-22

**Authors:** Wesley D Maciel, Alessandra C Faria-Campos, Marcos A Gonçalves, Sérgio VA Campos

**Affiliations:** 1 Bioinformatics PhD Program of the Universidade Federal de Minas Gerais, Belo Horizonte, 31270-901, Brazil; 2 Computer Science Department of the Universidade Federal dos Vales do Jequitinhonha e Mucuri, Diamantina, 39100-000, Brazil; 3 Computer Science Department of the Universidade Federal de Minas Gerais, Belo Horizonte, 31270-901, Brazil

## Abstract

**Background:**

Biological systems are commonly described as networks of entity interactions. Some interactions are already known and integrate the current knowledge in life sciences. Others remain unknown for long periods of time and are frequently discovered by chance. In this work we present a model to predict these unknown interactions from a textual collection using the vector space model (VSM), a well known and established information retrieval model. We have extended the VSM ability to retrieve information using a transitive closure approach. Our objective is to use the VSM to identify the known interactions from the literature and construct a network. Based on interactions established in the network our model applies the transitive closure in order to predict and rank new interactions.

**Results:**

We have tested and validated our model using a collection of patent claims issued from 1976 to 2005. From 266,528 possible interactions in our network, the model identified 1,027 known interactions and predicted 3,195 new interactions. Iterating the model according to patent issue dates, interactions found in a given past year were often confirmed by patent claims not in the collection and issued in more recent years. Most confirmation patent claims were found at the top 100 new interactions obtained from each subnetwork. We have also found papers on the Web which confirm new inferred interactions. For instance, the best new interaction inferred by our model relates the interaction between the adrenaline neurotransmitter and the *androgen receptor* gene. We have found a paper that reports the partial dependence of the antiapoptotic effect of adrenaline on *androgen receptor.*

**Conclusions:**

The VSM extended with a transitive closure approach provides a good way to identify biological interactions from textual collections. Specifically for the context of literature-based discovery, the extended VSM contributes to identify and rank relevant new interactions even if these interactions occcur in only a few documents in the collection. Consequently, we have developed an efficient method for extracting and restricting the best potential results to consider as new advances in life sciences, even when indications of these results are not easily observed from a mass of documents.

## Background

In a biological system there are entities of different types such as diseases and drugs performing important biological activities. The action of an entity can mediate or interfere with the action of other entities developing a complex network of interactions. Frequently entities perform more than one activity in the system, some which are known and integrate the current knowledge in life sciences. Other activities are not so well documented or remain unknown for long periods of time and are generally discovered by chance. Drugs, for instance, have a primary pharmacological activity and secondary activities responsible for side effects. However, drug side effects can be explored as new uses for the treatment of different diseases. A remarkable example is the impotence drug sildenafil citrate (Viagra®) that was originally designed for the treatment of angina and hypertension. Viagra® clinical trials revealed, nevertheless, the drug ability of increasing erectile function as its side effect [1].

On the other hand, research achievements in the post genomic age have promoted an enormous and continuous increasing on biological knowledge. These achievements often describe biological entity activities and have been published around the world aiming to assist, increase and speed up the number of discoveries in life sciences. A similar process has occurred since the inception of the World Wide Web and the rise of digital libraries. Web pages have been continuously and rapidly published given rise to a enormous amount of interlinked information. This allowed the conduction of many studies about methods for extracting and analysing the information published in this ocean of information. In many of these studies the vector space model (VSM) [2-4] has been recognized as an important tool to extract the most relevant information in a given context.

In this work we have developed an inference model based on the VSM in order to predict new interactions between biological entities of distinct categories such as ecosystems, organisms, organs, tissues, cells, organelles, genes, proteins, diseases and drugs. Our model constructs a network of known entity interactions from a textual collection. The documents in this collection describe the current knowledge in life sciences. Known entity interactions represent entity co-occurrences in at least one document of the textual collection. After finding all known interactions, our model traverses and analyzes the network predicting new entity interactions. Our objective is to use the known interactions to infer new (unknown) ones and to rank all found interactions. The ranking of interactions allows researchers to focus in the most promising activities, thus promoting further advances in life sciences.

The prediction of new interactions is performed using the VSM along with a transitive closure similar to that used in literature-based discovery [5]. The transitive closure relies on the fact that “**IF** an entity *x* interacts with entities *y* and *w ***AND** another entity *z* also interacts with entity *y*, **THEN ***z* probably also interacts with entity *w*”. Different from previous work, in our model we adapt this transitive closure in order to exploit the primary and secondary activities performed by entities of distinct biological categories. In the context of our model, *x* and *z* are entities of the same biological category, *y* and *w* are also entities of the same biological category. However, the category of entities *x* and *z* is different of that of entities *y* and *w*.

We have implemented a system called *BioSearch *[6] as a proof of concept of our model. The system deals with 4 types of distinct entities: diseases, drugs, genes, and targets. The textual collection used in the system encompasses a sample of 17,830 patent claims gathered from the United State Patent and Trade Mark Office (USPTO) [7]. We have used the patent claim because it is an important section in patent specifications, presenting the invention and defining the scope of patent protection [7,8]. From 266,528 possible interactions between entities in our network, the system has found 1,027 known interactions in the patent claim collection and has inferred 3,195 new interactions. Thus, based on our model, the system has constructed a network with 4,222 interactions that can be further analyzed in order to promote new advances in science and technology.

To validate our results we have conducted an experiment over the patent issue dates. We have reconstructed the interaction network in a range of 30 years. We have observed that new interactions found in a given past year were confirmed by patents issued in a more recent date. For instance, we have 1 patent claim issued in 2005 specifying the interaction between the disease heart attack and the gene *ppar-gama*. When we removed this patent claim from the textual collection, 61 patent claims indicated this interaction as a possible new interaction in 2004. We have also found scientific papers that confirm some of the new inferred interactions. For instance, the best result found in our model specifies a new interaction between the adrenaline neurotransmitter and the *androgen receptor* gene in the 2-dimensional subnetwork *gene* × *target*. No patent claim in our collection indicates this interaction. However, Sastry et al. [9] reported in 2007 that the antiapoptotic effect of adrenaline partially depends on *androgen receptor*.

### Related work

In this work, our objective is to present a model that employs the VSM in order to identify biological entity activities from a textual collection. In our approach, known entity activities represent entity co-occurrences in the textual collection. On the other hand, new activies are predicted from the known ones. Jenssen et al. [10] show that co-occurrence reflects biologically meaningful relationships, thus providing an approach to extract and structure known biological knowledge. Accordingly, we have developed a strategy based on the VSM that constructs a network of biological entity interactions from the life science literature and ranks these interactions. Our strategy combines the VSM ability to extract knowledge from text along with some underlining principles of literature-based discovery [11]. Don R. Swanson has pioneered the work in the field of literature-based discovery using the syllogism *x* → *y* AND *y* → *z* THEN *x* → *z* in order to discover new biological entity activities [12,13]. In this syllogism *x* → *y* and *y* → *z* are known interactions stated in the literature. On the other hand, *x* → *z* is a new interaction not explicitly found in the literature and inferred from previously known interactions. Afterwards, Smalheiser et al. [14] have implemented this syllogistic construction in a software called ARROWSMITH. In addition, Weeber et al. [15] have contributed to literature-based discovery introducing a model based on natural language processing (NLP) techniques in order to find concepts in the biomedical literature and reduce the search space. None of these techniques associates weights with these biological interactions in order to rank them.

As mentioned, a challenge we face when dealing with literature-based discovery is how to rank a large number of inferred interactions in a way that can facilitate new discoveries by prioritizing the ones with the largest potential. In order to tackle this challenge, Swanson et al. [16] have proposed and tested strategies to rank and filter the output of the ARROWSMITH system. Hristovski et al. [5,17-19] presented a method for literature-based discovery based on association rules and implemented it in a system called BITOLA. Moreover, Wren et al. [20] have considered the construction of networks from the biomedical literature describing a method based in the syllogism proposed by Swanson. They have defined areas of research interest such as genes and diseases, and model and rank the interactions using the fuzzy set theory. The ranking strategies used in these works consider that entities co-occurring frequently in a textual collection are more likely to represent biologically meaningful relationships [10]. Therefore, these strategies promote new interactions which are predicted from a large number of indications. However, in literature-based discovery there are many distinct scenarios and in some situations a great number of indications may not reveal the most relevant new interactions. For instance, many indications may lead to a set of new interactions that were already studied but were not published because they are not feasible or they are unwanted in practice. On the other hand, there exist situations in which new interactions predicted from a few number of indications are in fact the ones with the best potential. In this sense, ranking strategies for new interactions predicted from few indications are an important tool for literature-based discovery because they help in the identification of relevant interactions not easily observed and extracted from textual collections. In this scenario the VSM provides a great aid to the literature-based discovery. The TFIDF weighting strategy exploited in the VSM promotes interactions with many occurrences in few documents in the collection and penalizes interactions commonly occurring in many documents of the collection. Consequently, the VSM fosters rare interactions over the trite ones.

In literature-based discovery we must avoid the inference of interactions already stated in the literature. Kostoff [21,22] has discussed this problem and issues related to the quantity and quality of interactions. Kostoff et al. [23] have presented a generic methodology for literature-based discovery and have used this methodology to identify interactions concerning Raynaud’s phenomenon [24], cataracts [25], Parkinson’s disease [26], multiple sclerosis [27] and water purification [28]. Kostoff et al. [29] have also compiled the lessons learned in these experiments and presented guidelines for further research. However, in this series of works the authors have not used any numerical filter to rank the new interactions found.

We have also to cope with the coverage problem when looking for biological entity activities by searching several information sources such as experimental data [30,31], drug labels [32], scientific papers [5,12-20] and patents. Patents are very important instruments of knowledge transfer and researchers commonly resort to this literature because its great value as a source of strategic, technical and business-related information [33,34]. Trippe [35], for example, described *patinformatics* as the science of analyzing patent information to discover relationships and trends. Mukherjea et al. [36] developed a system to retrieve information from biomedical patents. Larkey [37] described the patent retrieval and classification system developed for the USPTO. Fall et al. [38] evaluated the best ways to deal with patent classification and presented a comparison of the classification effectiveness of several algorithms in this task. Tseng et al. [34] described and evaluated several text mining techniques to create patent maps and improve patent analysis tasks such as classification and knowledge sharing. Particularly, the claim section is considered the most important section in patent specifications [7,8]. Thus, Shinmori et al. [8] proposed a framework to represent the structure of the patent claim section and a method to automatically analyze it. Accordingly, here we also explore patents, more specifically, the patent claim section, along with our proposed model in order to discover new biological entity interactions of potential interest.

### Main contribution

We have created a model to construct networks of entity interactions from the biological literature with the objective of finding known and new entity activities in a biological system. In our model we have used VSM to identify already known entity interactions. In addition, we have extended the VSM with a transitive inference process capable of predicting new entity interactions. The networks are formed by subnetworks of interactions between entities of distinct categories. The advantage of using categories is the ability to restrict the research space for interactions between entities of specific categories and promote more accurate results.

Interactions are initially established in a network by entity co-occurrences in a textual collection. These interactions represent known interactions already described in the literature. The known interactions receive a weight corresponding the interaction level between entities based on the similarity value derived from the application of the VSM. The advantage of using the VSM is to explore its well documented algebraic framework for information retrieval from textual collections in order to find the entity co-occurrences and also measure their interaction levels. The VSM contributes for literature-based discovery by helping to predict the best new potential interactions not easily extracted from textual collections. The VSM also helps in situations in which entities rarely co-occurring in a document set are the ones with the potential best contributions for a researcher.

Our model uses the interactions established in the network to predict new interactions based on the transitive closure that we have employed in the inference process. The transitive closure states that “**IF** an entity *x* interacts with entities *y* and *w ***AND** an entity *z* interacts with entity *y ***THEN ***z* may also interact with *w*”. Differently from previous work, entities satisfying the transitive closure must always follow a constraint. The constraint imposes that *x* and *z* are entities of the same biological category *C*_1_, *y* and *w* are entities of another category *C*_2_, and that *C*_1_ and *C*_2_ are distinct categories (*C*_1_ ≠ *C*_2_). This constraint gives rise to the subnetworks that form the network of interactions. The main advantage of using this constraint is to narrow the research space of entity interactions promoting more accurate results. New interactions also receive a value for their interaction levels, based on the interaction levels of the interactions satisfying the transitive closure, as will be detailed later. This makes it possible to rank all entity activities in the network. The main advantage of ranking the network interactions is to reduce the human effort spent in their analysis, by focusing in the ones with the largest potential.

We have implemented the model in a system called *BioSearch* which uses a textual collection formed by patent claims. In our system, users can search all interactions established in the network (Appendix A [additional file]). Searching known entity interactions, users have a representation of the prior knowledge in a given subject that can be extracted from patent literature. These interactions are very important because they present a description of the current knowledge, avoiding patent infringements. On the other hand, users can search new interactions and have a representation of possible new technologies that may yet receive patent protection.

In the present work our goal is not to ensure a comprehensive coverage of the biological literature. Instead, we provide a proof of concept demostrating the applicability of our model in disclosing and ranking new entity interactions. For this, we have used a small textual collection to assess the model. Many new interactions inferred by our model based on this collection may have already been reported in scientific papers, thus validating our results.

## Network construction

**Figure 1:**
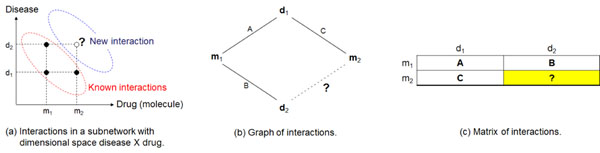
Representations of entity interactions in a 2-dimensional subnetwork.

Entities in a biological system interact with each other forming an interaction network. We can classify these entities into categories such as diseases, drugs, genes, and targets. In this work we have combined these categories in order to construct a network composed of n-dimensional subnetworks. We have extracted all entity interactions of a subnetwork from a textual collection using the VSM. Given, for instance, the subnetwork with dimensional space *drug* × *disease*, consider that our model indicates we have documents reporting the use of drug *m*_1_ in the treatment of diseases *d*_1_ and *d*_2_ (Figure 1 (a) ). Moreover, suppose the model also indicates we have documents which report the use of drug *m*_2_ in the treatment of disease *d*_1_. Then, drugs *m*_1_ and *m*_2_ possibly share some common characteristic responsible for the efficacy of these drugs in the treatment of both diseases *d*_1_ and *d*_2_. Thus, the model infers a new connection in the subnetwork *drug* × *disease* linking drug *m*_2_ and disease *d*_2_. The new connection represents a new use of drug *m*_2_. Then, in this example, *m*_1_ → *d*_1_, *m*_1_ → *d*_2_, and *m*_2_ → *d*_1_ are known interactions found in the literature. On the other hand, *m*_2_ → *d*_2_ is a new interaction inferred from the previous three known interactions.

We have represented each subnetwork as a weighted graph whose weights measure the interaction level of the entities based on the textual collection. In this graph, nodes are entities of categories forming the subnetwork dimensional space, edges represent interactions between entities of distinct categories, and the interaction level is a value in the range [0, 1]. We determine the interaction level based on the VSM when we look for the entity co-occurrences throughout the textual collection. In a subnetwork with dimensional space *drug* × *disease*, for instance, suppose that drug *m*_1_ treats diseases *d*_1_ with interaction level *A* and *d*_2_ with interaction level *B* (Figure 1 (b)). In addition, suppose drug *m*_2_ treats disease *d*_1_ with interaction level *C*. Then, the model assigns an interaction level to the new connection linking drug *m*_2_ and disease *d*_2_ whose value is determined based on *A*, *B* e *C*.

The graph in our model is represented by a matrix that receives biological entities of the subnetwork dimensions in its lines and columns (Figure 1 (c)). We have defined that three interactions in the matrix are in transitive closure when they satisfy the condition (*x*, *y*) *and* (*x*, *w*) *and* (*z*, *y*) → (*z*, *w*) that means ”**IF** entity *x* interacts with entities *y* and *w ***AND** entity *z* interacts with entity *y ***THEN ***z* may also interact with *w*”. Then, the model infers a new interaction in the matrix whenever it finds three interactions satisfying this transitive closure.

**Figure 2:**
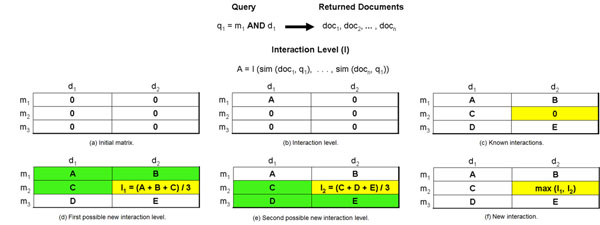
Network construction.

All cells in the matrix initially receive the value 0 indicating no entity interactions (Figure 2 (a)). We use the entities of a cell in order to form a query. This query represents a conjunction of entities of distinct categories. The conjunction is important because it ensures that documents in which the entities occur are not orthogonal, i.e., they must have occurrences of all entities present in the query. Then, we perform searches in the textual collection in order to find documents satisfying the query of each matrix cell (Figure 2 (b) ).

The VSM assigns weight values for each query entity based on the TFIDF strategy (Equation 1). We use these weights to measure the importance of the entity for a query of the matrix and also for a document of the textual collection.(1)

In the TFIDF weight strategy *w*_*x*,*i*_ is the weight of entity *e_x_* in a document *d_i_* in the textual collection, *tf*_*x*,*i*_ is the normalized frequency of entity *e_x_* in document *d_i_*, *idf_x_* is the inverse document frequency of entity *e_x_*, *f*_*x*,*i*_ is the frequency of entity *e_x_* in document *d_i_*, *max*_*j*,*i*_ is the number of times the most frequent entity *e_j_* occurs in document *d_i_*, *N* is the number of documents in the textual collection, and *n_x_* is the number of documents in the textual collection in which entity *e_x_* occurs.

Each query of the matrix receives a similarity value for each document in the textual collection based on the VSM (Equation 2). For the VSM similarity, *q_j_* is a query *j* of the matrix representing a conjunction of entities, *t* is the number of biological entities of the network, *w*_*x*,*i*_ is the weight of entity *e_x_* in document *d_i_*, *w*_*x*,*j*_ is the weight of entity *e_x_* in query *q_j_*. In our model, the entity weights in a query are always 1 (*w*_*x*,*j*_ = 1). The similarity value stated in the VSM indicates the relevance of a document for a query.(2)

We use the similarities returned by equation 2 to determine the interaction level of the query entities. The cell linking the query entities receives this interaction level which represents a known interaction in the subnetwork (Figure 2 (b)). In our current experiments we determine the interaction level of a known interaction in 3 different ways: (i) the arithmetic average of the similarities, (ii) the maximum similarity found, and (iii) the sum of all the similarities.

After all searches in the textual collection are concluded, we have established all known interactions of the network. However, some cells remain equal to 0 indicating that some entity interactions are not explicitly mentioned in the collection (Figure 2 (c)). These cells with value 0 represent the potential new interactions between the biological entities they relate.

The model infers a new interaction in the matrix whenever it finds three interactions satisfying the transitive closure (Figures 2 (d) and 2 (e)). In our current experiments the interaction level of a new interaction is the arithmetic average of the interaction level of the three interactions satisfying the transitive closure. If many interactions satisfy the transitive closure, the model chooses the one with highest arithmetic average (Figure 2 (f)).

We have applied several iterations of our model on the matrix of a subnetwork in order to infer new interactions from interactions previously inferred. In iteration 0 the model discovers all known interactions reported in the textual collection. In iteration 1 the model discovers new interactions based on the known interactions. In iteration 2 the model discovers new interactions based on interactions discovered in iterations 0 and 1. The model stops iterating when all cells of the matrix receives a value different from 0 or when it is no more possible to find interactions satisfying the transitive closure. Starting from iteration 1, our model divides the interaction level of new interactions by the number of iterations performed. This penalty ensures that interactions found in earlier iterations have higher interaction levels.

## Methods

In our experiments, we have considered a sample of patent claims crawled from the USPTO Web site constituting a textual collection with 17,830 documents. All these patents were issued between 01/01/1976 and 12/31/2005. Besides, in the claim section of all these patents we are able to find at least one entity of the four biological categories considered in our crawling process, namely diseases, drugs, genes, and targets. In the USPTO Web site the query we have used to retrieve these patents is represented as *aclm/”entity” and isd/1/1/1976* → *31/12/2005* where *aclm* specifies the patent claim section, *entity* is the biological entity name, and the *isd* specifies the patent issue date, respectively. The entity names are quoted in order to specify the phrase search mode.

As mentioned, we have considered entities of 4 biological system categories: diseases, drugs, genes, and targets (Table 1). We have chosen these categories based on their importance for life sciences research and the practical applications of their entity interactions for the society. The category disease corresponds to a set of possible states of a biological system (e. g. breast cancer, type 2 diabetes, and atherosclerosis). The category drug corresponds to a set of molecules capable of changing the state of a biological system (e. g. aspirin, diclofenac, and tamoxifen). The categories gene and target correspond to a set of building blocks of the biological system. The category gene is a set of building blocks responsible for generating other building blocks (e. g. *major histocompatibility complex class I*, and *tumor suppressor p53*). The category target is a set of building blocks generated by genes and over which a drug acts (e. g. cachectin, and progesterone receptor).

**Table 1:** Categories and web sources of the biological entities.

Category	Number of Entities	Number of Clusters	Web Source
Disease	52	22	Karolinska Institute [45], Mayo Clinic [46], Therapeutic Target Database [47], Drug Bank [48], Medline Plus [49]
Drug	44	22	Drugs.com [50], Patient.uk [51], Therapeutic Target Database, Drug Bank
Gene	43	20	Kyoto Encyclopedia of Genes and Genomes [52], HUGO Gene Nomenclature Committee [53], NCBI Entrez Gene [54]
Target	50	23	The Free Dictionary [55], Therapeutic Target Database, Drug Bank

Total	189	87	

In order to detect the entity occurrences throughout the collection, we have used exact string matching over the entity names and we have also considered entity related names such as synonyms. For instance, we have considered diabetes mellitus type 2 and type 2 diabetes as the same biological entity of category *disease*. We have formed clusters of related names for each entity (Appendix B [additional file]). A representative single name in each cluster is used to represent the whole cluster during the network construction. Some syntactic variation in entity names are also considered in each cluster (e. g. Alzheimer’s disease and Alzheimer disease).

In our experiments all categories forming a subnetwork are disjoint sets. For instance, the categories gene and target do not have entities in common when forming the subnetwork *gene* × *target*. Combining these 4 biological categories, we have a network composed by 11 subnetworks (Table 2). Of these, 6 have 2 dimensions, 4 have 3 dimensions, and 1 has 4 dimensions.

**Table 2:** The subnetworks.

Subnetwork	Dimensional Space
1	*disease* × *drug*
2	*disease* × *gene*
3	*disease* × *target*
4	*drug* × *gene*
5	*drug* × *target*
6	*gene* × *target*

7	*disease* × *drug* × *gene*
8	*disease* × *drug* × *target*
9	*disease* × *gene* × *target*
10	*drug* × *gene* × *target*

11	*disease* × *drug* × *gene* × *target*

In the current implementation of our model we neither use natural language processing (NLP) [5,15] nor heuristics to capture the context in which the entity names are applied in the documents. Notwithstanding, the entity names we have selected were satisfactory for our purpose of validating the model, as we shall see.

## Results

### Network construction

In our experiments the biological network has 266,528 possible interactions. Searching the patent claim collection our model has identified 1,027 known interactions (Table 3). Based on these known interactions our model was able to infer 3,195 new interactions.

**Table 3:** The subnetworks and their number of known and new interactions.

Subnetwork	Dimensional Space	Known Interaction	New Interaction	Total
1	*disease* × *drug*	192	270	462
2	*disease* × *gene*	76	184	260
3	*disease* × *target*	138	346	484
4	*drug* × *gene*	38	130	168
5	*drug* × *target*	105	294	399
6	*gene* × *target*	50	175	225

7	*disease* × *drug* × *gene*	71	304	375
8	*disease* × *drug* × *target*	199	958	1.157
9	*disease* × *gene* × *target*	55	269	324
10	*drug* × *gene* × *target*	34	76	110

11	*disease* × *drug* × *gene* × *target*	69	189	258

Total		1 027	3 195	4 222

We have ranked the subnetworks according to their best new interactions (Table 4). In most cases, subnetworks with few dimensions had the higher interaction levels. This happens because in a subnetwork with many dimensions it is more difficult to find documents in which entities of all dimensions co-occur. However, we find more accurate results in subnetworks with more dimensions because the model is able to better constrain the research space when we increase the number of dimensions of a dimensional space.

**Table 4:** Ranking of subnetworks based on their best new interactions and number of dimensions. The interaction level of known interactions was determined by the arithmetic average of all similarities returned by the vector space model.

Subnetwork	Dimensional Space	New Interaction	Level of Interaction
6	gene target	androgen receptor adrenaline	0.9757
2	disease gene	HIV transforming growth factor, beta 1	0.9738
5	drug target	verapamil cyclooxygenase 2	0.9597
1	disease drug	erectile dysfunction divalproex	0.9470
3	disease target	arrhythmia cyclic-gmp phosphodiesterase	0.9272
4	drug gene	ciclosporin androgen receptor	0.8211

8	disease drug target	alzheimer dementia acetylsalicylic acid adrenaline	0.8807
10	drug gene target	acarbose apolipoprotein a 1 lymphotoxin	0.8723
9	disease gene target	parkinson disease apolipoprotein e choline acetylase	0.8695
7	disease drug gene	gout hydrochlorothiazide endothelin 1	0.8357

11	disease drug gene target	breast adenocarcinoma tamoxifen ppar-gamma hmg-coa reductase	0.7826

### Validation

**Figure 3:**
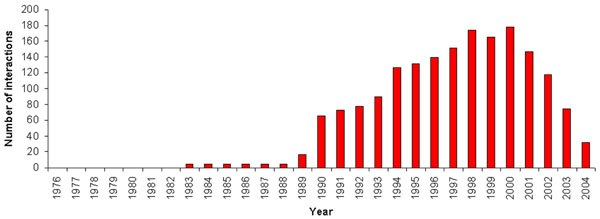
Interactions with confirmation patent claims by year.

Removing patent claims from our collection according to the years in which they were issued and applying our model after each remotion, we observed that new interactions found in a year were confirmed by patent claims removed from the collection and issued in more recent years (Figure 3). For example, in order to better assess the quality of our model, we have analyzed known interactions established in the network in 2005 that became new interactions in 2004 when the patent claims issued in 2005 were removed from our textual collection (Table 5). These known interactions in 2005 represent patents filed in 2005 that our model would have identified in 2004. Thus, we used these known interactions in 2005 as confirmation patent claims for new interactions inferred in 2004. For instance, the interaction between the disease heart attack and the gene *ppar*-*gama* has 1 patent claim issued in 2005. When we removed this patent claim from the collection, 61 patent claims indicated this interaction as a new one in 2004.

**Table 5:** The top 5 known interactions with high interaction level in 2005 that became new interactions in 2004. The interaction level of known interactions was determined by the arithmetic average of all similarities returned by the vector space model.

Subnetwork	Dimensional Space	Interaction	Level in 2005	Level in 2004	Patents in 2005	Patents in 2004
5	disease gene	heart attack ppar-gamma	0.9999	0.8324	1	61
1	target disease	adrenaline cardiac ischemia	0.9866	0.8676	1	36
11	target disease drug gene	hmg coa reduct. breast cancer tamoxifen kennedy disease	0.9190	0.6383	1	5
2	target drug	gp iib/iiia neoral	0.9137	0.8354	1	103
4	disease drug	HIV bonyl	0.9041	0.8825	1	30

**Figure 4:**
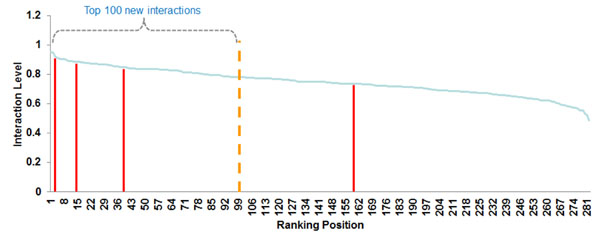
**Distribution of confirmation patent claims filed in 2005 throughout the levels of the ranking constructed with patents issued up to 2004 for the subnetwork *drug* × *target***. The number of new interactions predicted in this subnetwork in 2004 is 282. We have found 4 confirmation patent claims filed in 2005 for the new interactions predicted in 2004. The position of these 4 confirmation patent claims in the ranking of new interactions predicted in 2004 are respectively 3, 14, 39, and 159. Thus, we have observed that 3 confirmation patent claims were among the top 100 best ranked indications of subnetwork *drug* × *target*.

Removing all patent claims issued in 2005, our model predicted 2,930 new interactions based on patents issued up to 2004. Among these new interactions in 2004, we had 32 confirmation patent claims filed in 2005. We then verified the top 100 new interactions found in 2004 for each subnetwork in order to check whether these 32 confirmation patents were among the highest ranked indications of our method (Figure 4).

We have observed the distributions of confirmation patent claims when the known interactions were determined by the average, maximum and sum strategies applied over the similarities returned by the VSM. In the subnetwork *disease* × *drug*, for instance, we had 275 new interactions in 2004 (Table 6). This subnetwork had 5 new interactions with confirmation patent claims issued in 2005. When we used the arithmetic average strategy for known interactions we had 3 new confirmed interactions at the top 100 new interactions of this subnetwork ranking. On the other hand, with the maximum and sum strategies we found 4 new confirmed interactions at the top 100 new interactions of this subnetwork. Further, the first confirmed new interaction in this subnetwork is among the top 10 interactions of the ranking and the second one is among the top 20 when we used the arithmetic average strategy. In sum, when we applied the average, maximum and sum strategies, we found 53%, 56%, and 69% of the 32 confirmation patents at the top 100 new interactions of all subnetworks, respectively.

**Table 6:** The subnetworks and their number of confirmation patent claims at the top 100 new interactions predicted in 2004.

Subnetwork	Dimensional Space	New Interactions in 2004	Confirmations Issued in 2005	Distribution at the Top 100 New Interactions		
				AVG	MAX	SUM
1	*disease* × *drug*	275	5	3	4	4
2	*disease* × *gene*	167	2	2	2	2
3	*disease* × *target*	348	2	1	1	1
4	*drug* × *gene*	119	0	0	0	0
5	*drug* × *target*	282	4	3	3	3
6	*gene* × *target*	152	3	2	2	3

7	*disease* × *drug* × *gene*	308	4	1	1	4
8	*disease* × *drug* × *target*	786	9	2	2	3
9	*disease* × *gene* × *target*	242	2	2	2	2
10	*drug* × *gene* × *target*	76	0	0	0	0

11	*disease* × *drug* × *gene* × *target*	175	1	1	1	0

Total		2 930	32	17	18	22

%				53	56	69

In addition, we have looked for papers on the Web in order to confirm some of the new interactions found in 2005. For instance, the best result found in our model relates the interaction between the *androgen receptor* gene and the adrenaline neurotransmitter in the 2-dimensional subnetwork *gene* × *target*. Sastry et al. [9] reported that the antiapoptotic effect of epinephrine partially depends on androgen receptor. A modest decrease in the antiapoptotic effect of epinephrine in cells where *androgen receptor* expression was reduced provides evidence that epinephrine reduces sensitivity of cancer cells to apoptosis.

We have found confirmation papers on the Web for the first new interaction of five 2-dimensional subnetworks (Table 7). Out of six 2-dimensional subnetworks, four have had their most relevant new interaction confirmed by later papers issued from 2007 up to 2009. In just one case we have not found a confirmation paper for the first new interaction, in the ranking of the 2-dimensional subnetwork *drug* × *target*. We have not found any confirmation paper for the first new interaction in the ranking of the 3-dimensional subnetworks neither for the 4-dimensional subnetwork. We have not looked for papers on the Web for new interactions in other positions of the rankings in each subnetwork.

**Table 7:** Confirmation papers for the first new interaction predicted in 2005 for each subnetwork.

Subnetwork	Dimensional Space	First Interaction	Confirmation Papers
1	*disease* × *drug*	impotence divalproex	[56,57]
2	*disease* × *gene*	acquired immunodeficiency syndrome transforming growth factor, beta 1	[58,59]
3	*disease* × *target*	arrhythmia cyclic-gmp phosphodiesterase	[60]
4	*drug* × *gene*	ciclosporin androgen receptor	[61,62]
5	*drug* × *target*	verapamil cyclooxygenase 2	none
6	*gene* × *target*	androgen receptor adrenaline	[9]

7	*disease* × *drug* × *gene*	gout hydrochlorothiazide endothelin 1	none
8	*disease* × *drug* × *target*	alzheimer’s disease aspirin adrenaline	none
9	*disease* × *gene* × *target*	parkinson’s disease apolipoprotein e choline acetylase	none
10	*drug* × *gene* × *target*	acarbose apolipoprotein a-1 lymphotoxin	none

11	*disease* × *drug* × *gene* × *target*	breast cancer tamoxifen ppar-gamma hmg-coa reductase	none

### Example session

#### Research space of new interactions

**Figure 5:**
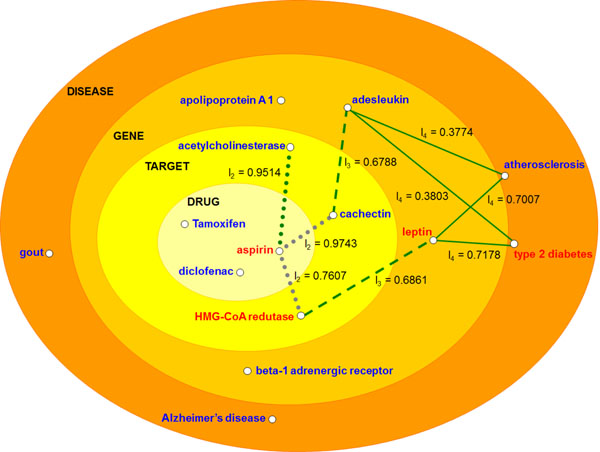
**Research space for some possible interactions with the drug aspirin**. Gray lines are known interactions and green lines are new interactions. The interaction level of known interactions was determined by the arithmetic average of all similarities returned by the vector space model.

In our model, subnetworks with more dimensions constrain better the search space for new interactions, thus promoting more accurate results. For instance, consider a researcher using our system who is interested in new interactions related to the drug aspirin. Initially, the researcher decides to analyze the interactions of aspirin with HMG-CoA reductase, cachectin and acetylcholinesterase targets (Figure 5).

Our system shows that the best option would be to conduct research about the interaction between aspirin and acetylcholinesterase, since this interaction has a high interaction level (*l*_2_ = 0.9514 where *l_n_* is the interaction level in the *n*-dimensional subnetwork, *n* = 2, 3, 4...) and the other two are known interactions. Therefore, our model predicts the interaction between aspirin and acetylcholinesterase as a very promising research topic. However, the researcher can still reach more precise results since the search space is still very large and these entities can interact with several other entities of distinct categories. In other words, the researcher may obtain even more accurate results when considering subnetworks with more dimensions.

Using a 3-dimensional subnetwork, the researcher now considers the dimension gene in the analysis. Then, the researcher discovers that the interaction between aspirin and the acetylcholinesterase becomes less promising because no interaction between these entities is established in the 3-dimensional subnetwork. The researcher realizes that in the 3-dimensional subnetwork *drug* × *gene* × *target* the interaction between aspirin, HMG-CoA reductase and *leptin* with interaction level *l*_3_ = 0.6861 becomes the most promising research topic. Finally, going a step further by searching the 4-dimensional subnetwork, the researcher discovers that the interaction among aspirin, HMG-CoA reductase, *leptin* and type 2 diabetes with interaction level *l*_4_ = 0.7178 is in fact the most promising interaction for research.

#### Interaction history

**Figure 6:**
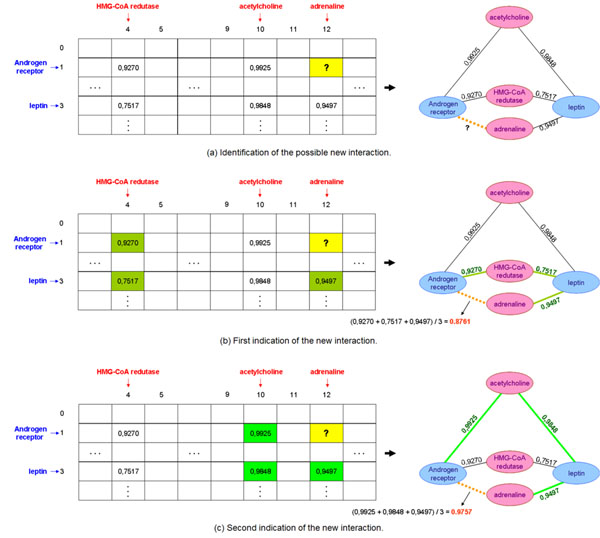
**History of the inference process**. Entities in blue are genes and entities in red are targets. The interaction level of known interactions was determined by the arithmetic average of all similarities returned by the vector space model.

The history of how each new interaction may have been established in the network can be followed with the *BioSearch* system. As an example, we observe the history of the new interaction with highest interaction level in the network when we used the arithmetic average strategy to determine the known interaction values. Our model inferred this interaction in 3 steps on the matrix of subnetwork *gene* × *target* (Figure 6).

**Figure 7:**
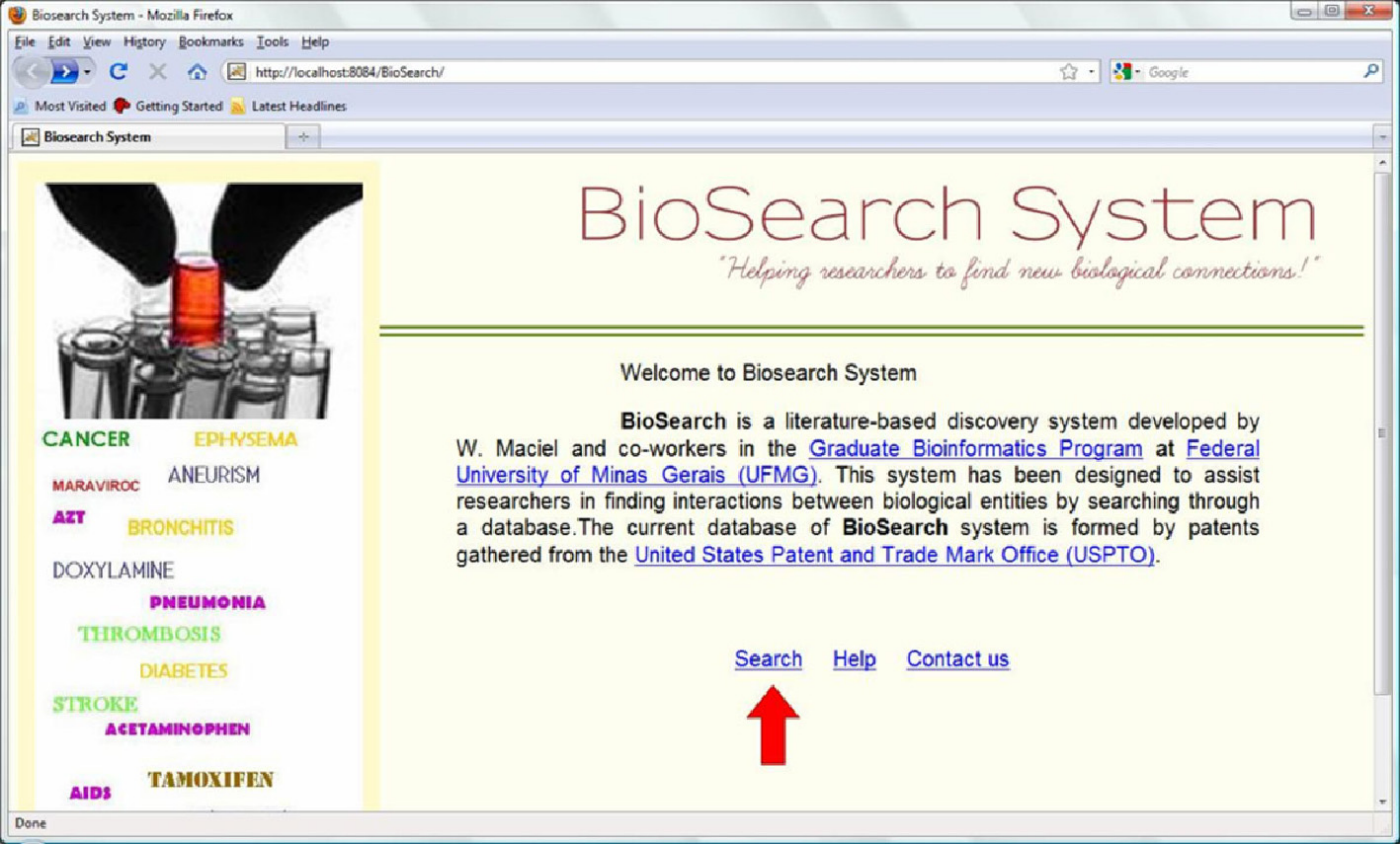
Biosearch system home page.

**Figure 8:**
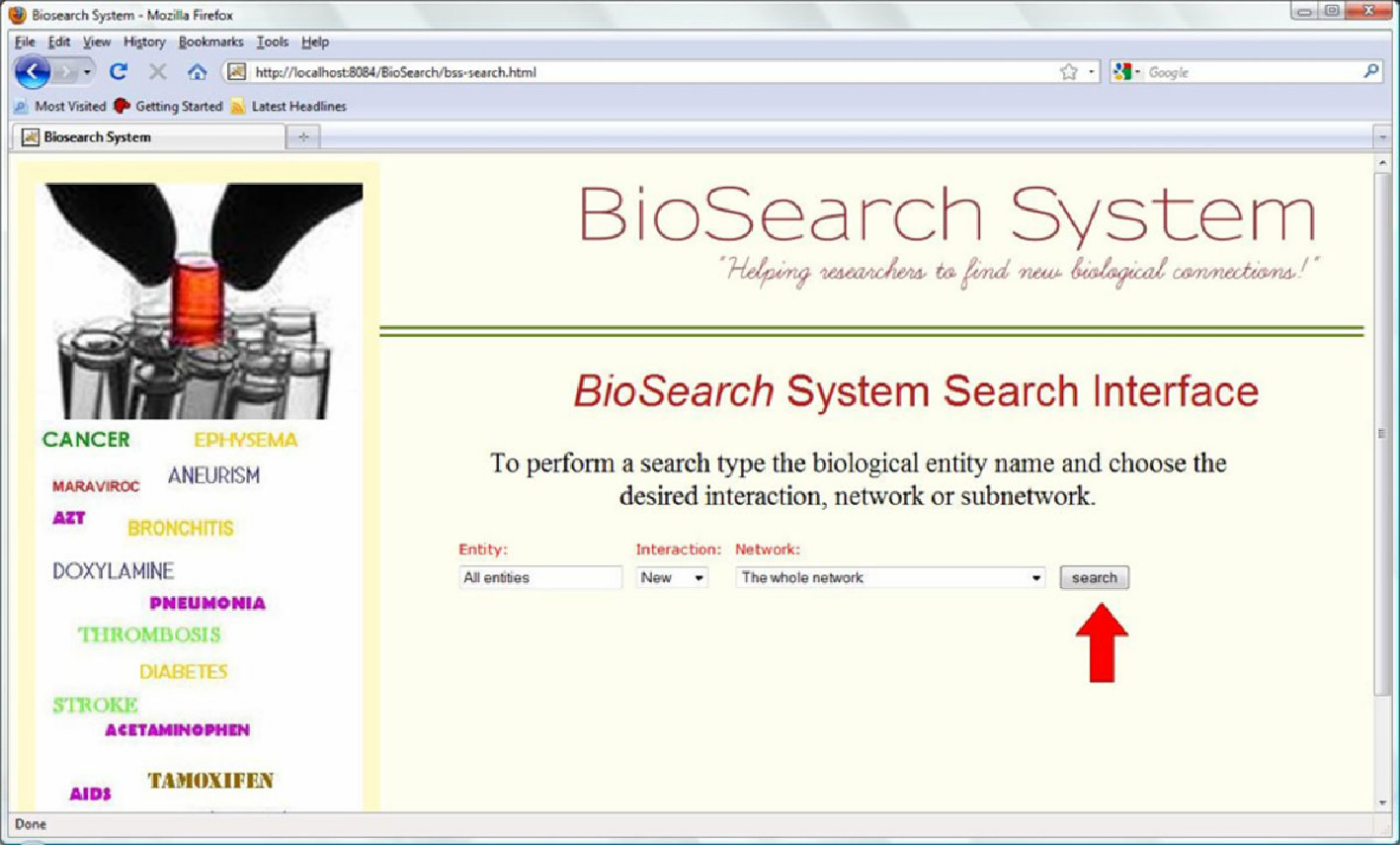
Biosearch system search page.

**Figure 9:**
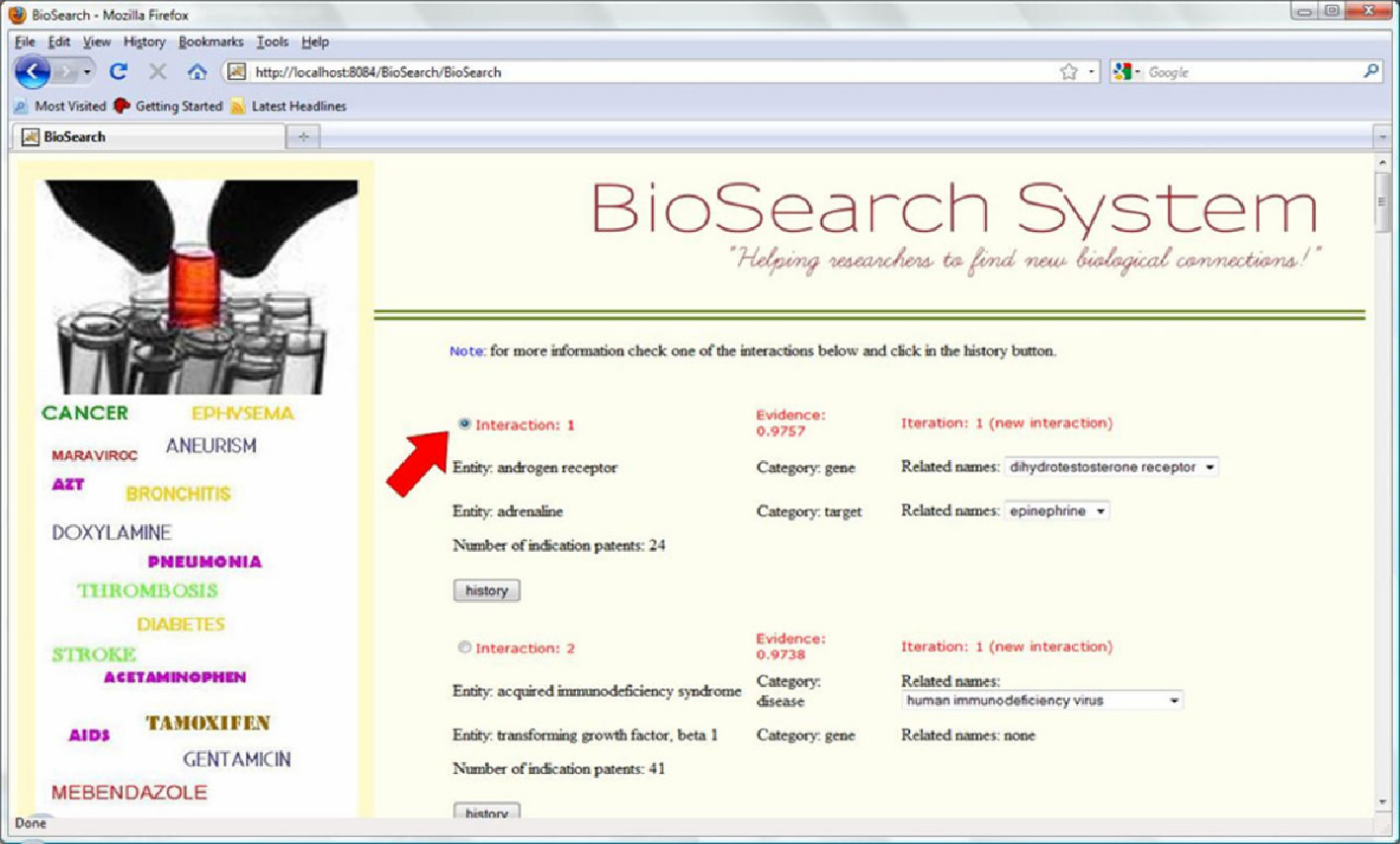
Biosearch system ranking page.

In the first step the model identifies the possible new interaction between the *androgen receptor* gene and the adrenaline target (Figure 6 (a)). In the second step, the model finds three known interactions in the transitive closure. These known interactions produce an interaction level for the new interaction with value 0.8761 (Figure 6 (b)).

In the third step, the model finds other three known interactions in the transitive closure. In this case, the known interactions produce a new interaction with value 0.9757 (Figure 6 (c)). No more possibilities are found for this new interaction. Thus, the interaction level found at the third step becomes the interaction level of the new interaction because it is higher than that found in the second step.

## Discussion

We have been able to achieve significant results in a strategy that combines the VSM with an inference process in order to predict new biological entity activities. We have used this strategy to model biological systems and to construct a network of biological entity interactions. Modeling biological systems is a complex task for many reasons. For example, we must consider a large number of biological parameters, we must identify entity concentrations and roles in different reactions, and we must bear in mind that biological systems are not linear systems and perturbations commonly give rise to unexpected results. Thus, we have abstracted details and studied biological systems in a higher level in order to decrease their complexities and conduct our analysis [39]. Our abstraction of biological systems is constructed from textual collections that represent a particular view of the technological advances in life sciences reported in patent claims.

In our model, we have focused on retrieving biological entity information from a textual collection consisting of patent claims using the VSM and expressing this information in a transitive closure. This approach has allowed several analysis with important findings. The approach has indicated the VSM as a useful tool to retrieve relevant information in an inference process and how the biological knowledge is interconnected in patent claims.

Texts in patents have a particular writing style characterized by a rich technical terminology and an intentional vagueness in order to promote wide protection to inventions [8,33,34,36,38]. This intentional vagueness may bring a potential benefit to our inference strategy of new interactions. The vagueness in patent texts can indicate some known interactions not easily observed in other literatures characterized by a more strict writing style, as scientific papers. From these known interactions we can infer new ones that are even more innovative than those predicted from texts with strict writing style.

As observed in some contemporary search engines, term co-occurrences is a good way to restrict the documents which can better satisfy an user information need. Thus, term co-occurrences is a good strategy to isolate good hits from a big mass of documents. In addition, previous work in the field of literature-based discovery have indicated many important findings relying on term co-occurrences [12,13,16]. Particularly, Jenssen et al. [10] have shown that co-occurrences reflect biologically meaningful relationships, thus providing an approach to extract and structure known biological knowledge. Accordingly, in our work we have relied on term co-occurrences in order to produce relevant results from a textual collection.

Our model has predicted 2,930 new interactions considering patent claims issued up to 2004. In 2005, we have 32 patent claims in which these new interactions are mentioned. These 32 patent claims issued in 2005 serve as confirmations for the new interactions predicted in 2004. We have also observed that using the VSM we have ranked up to 69% of these 32 new interactions among the 100 first new interactions of all subnetworks. In other words, 69% of the confirmed interactions would have been identified within the top 100 new indications of all subnetworks. These 32 confirmation patent claims also demonstrate that implicit interactions not easily observed in a textual collection must be recognized as important contributions in the field of literature-based discovery.

We consider the 32 new interactions with confirmations as a significant number mainly when considering the reduction in the number of biotechnological patents filed from 2001 to 2004 [40], and the fact that patents are not filed for the majority of scientific discoveries, being instead published as research papers [41-43]. In fact, we already expected a small number of confirmation patent claims for two main reasons. First due to the fact that these are patents filed in 2005, only one year after the new interactions were indicated by the collection issued up to 2004. A higher number of patents may have been filed in later years which would provide more indications. Second, filing a patent is the final step of a long sequence of activities related to scientific discovery, and most researchers stop in the scientific article publication phase. As such, a large number of confirmation patent claims should probably never been expected.

Our findings have encouraged us to further investigate biological parameters we have to use in order to improve our representation of biological systems and achieve better results in the inference process and ranking strategy. These parameters have another important function in preventing noise propagation. Interactions poorly established in the network propagate spurious interactions in the inference process. Thus, this study should help impose constraints to the identification of interactions during network construction. The definition of these parameters for several sources and their integration in our model is also an important concern.

In literature-based discovery, simple ranking strategies that promote new interactions based on the raw frequency of known interactions found in textual collections are often used. However, they show, in some situations, implicit interactions that have already been studied but were not documented because they are not feasible or are unwanted in practice. Our results demonstrate, on the other hand, that ranking strategies based on the VSM are good tools for the identification of significant implicit interactions occurring in texts, mainly those occurring in few documents of a textual collection. This is an important contribution because it is far more difficult to find relevant new interactions from knowledge not frequently co-occurring in a literature than that often observed. However, we must always keep in mind that relevance is a subjective concept. Therefore, biological entity interactions may be considered differently, i.e., with different importance, by different researchers. Then, we must consider strategies in literature-based discovery as complementary tools that help to identify the best new interactions based on the researcher’s interests. In this sense, we should even think of systems in which new ranking strategies may be integrated as add-ons.

We should also emphasize that our goal is not to ensure a complete coverage of the biological literature, creating an enormous network of known interactions. Instead, we focus in providing a proof of concept to show the VSM applicability to disclose and rank biological entity activities based on implicit connections found in biological literature. Accordingly, we have checked the existence of these implicit connections in patent claims using a small and restricted textual collection just for assessing the model. We are aware that many new interactions inferred by our model have already been reported in scientific papers. However, we have observed that these findings had not received patent protection at the USPTO until 2005 and we have used some of these scientific papers as validation of our results, mainly due to the inexistence of textual collections currently available for validating literature-based discovery systems [21,22,44]. For a production system we should index as much as possible of the current biological literature sources in order to filter prior art. Nevertheless, we have observed that our strategy provides a good tool for tracking scientific advances published in scientific papers but not yet protected under the intellectual property law.

## Conclusions

In this work we have introduced a technique that employs the Vector Space Model (VSM) for the identification of biological entity activities based on a network of biological entity interactions extracted from textual collections. The algebraic framework of the VSM has demonstrated to be a helpful tool in the task of finding known biological entity activities. We have extended the VSM with a transitive closure approach in order to predict new potential biological entity activities. The transitive closure we have used explores the primary and secondary activities of entities in a biological system. In addition, we have imposed a constraint in this transitive closure in order to ensure that interactions established in the network connect entities of distinct categories. This constraint reduces the search space for new interactions, promoting more accurate results. Moreover, we have used the similarity values derived from the VSM to rank the new discovered entity activities.

Our experiments using a collection of USPTO patent claims demonstrate that the biotechnological patent literature has implicit connections that can be explored to provide further advances in life sciences. Iterating our model according to the years in which the patent claims were issued, new interactions found in a year were confirmed by patent claims not in the collection and issued in more recent years. The experiments also showed that many confirmation patent claims were found for interactions at the top of our ranks of results. For instance, considering the ranking strategy based on the sum of the similarities returned by the VSM we had 69% of the confirmation patents among the first 100 new interactions of all subnetworks. We have also found scientific papers that validate several of the suggested interactions.

For future work we intend to construct networks using other patent fields (e.g. title, abstract and description sections), the whole patent text, and other sources, such as paper abstracts, paper titles and drug labels. We will analyze the contribution of all these pieces of evidence in our inference process when they are considered separately and together. In addition, we intend to explore natural language processing techniques and ontologies in order to improve the identification of entity co-occurrences in the textual collection. Moreover, we also want to conduct our analyses by considering entities co-occurring in one sentence, in a window of sentences, and in a whole paragraph in order to evaluate a phrase-based VSM approach in the context of our model. Then, we will apply proximity criteria for these occurrences in order to ensure the semantic interaction between entities. Furthermore, we will evaluate a set of biological parameters extracted from the literature in order to help with the establishment of interactions in the networks. Finally, we intend to study other possible strategies to rank biological interactions and conduct a trend analysis on how the interaction value evolves when restricting the number of documents in the textual collection.

## Competing interests

The authors declare that they have no competing interests.

## Authors' contributions

WDM, ACFC and SVAC conceived the project. ACFC and SVAC directed the project. WDM conceived and designed the model; implemented and tested the algorithms; prepared and tested the data. WDM and MAG conceived, designed and performed the computational experiment. ACFC and SVAC provided support throughout the research process. ACFC and SVAC hosted the BioSearch system in their lab at UFMG. All authors analyzed the data and results; and wrote, read and approved the final paper.

## Supplementary Material

Additional file 1 **Appendix.** A concise explanation of the Biosearch system interface and the clusters of entity names we have used in our current experiments. Click here for file
