# Supplementary material for: Can the vector space model be used to identify biological entity activities?
Source: BMC Genomics. 2011 Dec 22;12(Suppl 4):S1. doi: 10.1186/1471-2164-12-S4-S1 (PMC3287578; doi:10.1186/1471-2164-12-S4-S1)
Supplement: Additional file 1 — Appendix. A concise explanation of the Biosearch system interface and the clusters of entity names we have used in our current experiments. [file 1471-2164-12-S4-S1-S1.pdf]

Appendix

Appendix A: Biosearch system interface

In this appendix we present a concise explanation of the Biosearch system interface.

Appendix A:.1 Home page

In the home page of the Biosearch system the user will find a brief explanation about the system. In addition, the user can choose to search the system and visit the help or contact page (Figure 7).

Appendix A:.2 Search page

In the search page of the Biosearch system the user can express her information need as a query. In order to make a query the user can choose the name of an entity and the interaction type. In addition, the user can choose to performe her query in the whole network or in some of the subnetworks (Figure 8).

Appendix A:.3 Ranking page

The Biosearch system presents a ranking page with the interaction position, the interaction level (evidence), the interaction type, the entity names forming the interaction and the number of documents used to establish the interaction in the network (Figure 9).

Appendix B: Clusters of entity names

In this appendix we present the clusters of entity names we have used in our current experiments. The entity names are presented in their respective categories. In each category, a line represents a cluster. Entity names in a cluster are separeted by the semicolon character (;).

Category: disease

1. Diabetes mellitus type 2; noninsulin-dependent diabetes mellitus; type 2 diabetes; non-insulin-dependent diabetes mellitus.
2. Alzheimer disease; alzheimer dementia; alzheimer's dementia; alzheimer's disease; alzheimers disease.
3. Atherosclerosis.
4. Acquired immunodeficiency syndrome; human immunodeficiency virus; human immunodeficiency virus disease.
5. Breast cancer; cancer of the breast; cancer of breast; breast adenocarcinoma.
6. Gouty arthritis; gout.
7. Parkinson disease; parkinson's disease; parkinsonism; parkinsons disease.

8. Rheumatoid arthritis.
9. Lung cancer.
10. Cardiac ischemia.
11. Impotence; erectile dysfunction.
12. Thrombosis.
13. Arrhythmia; cardiac arrhythmia; dysrhythmia.
14. Epilepsy.
15. Ovarian cancer; cancer of the ovary.
16. Prostate cancer; cancer of the prostate; prostate carcinoma.
17. Pulmonary arterial hypertension; pulmonary hypertension.
18. Myocardial infarction; acute myocardial infarction; heart attack.
19. Postpartum depression; postnatal depression.
20. Dementia with lewy bodies; lewy body dementia; diffuse lewy body disease; cortical lewy body disease; senile dementia of lewy type.
21. Pernicious anemia.
22. Basal cell carcinoma.

Category: drug

1. Sildenafil citrate; viagra®.
2. Rosiglitazone.
3. Acarbose.
4. Rivastigmine.
5. Donepezil; donepezil hydrochloride; aricept®.
6. Sodium valproate; divalproex sodium; divalproex.
7. Carbamazepine.
8. Trazadone; desyrel®.
9. Atorvastatin; lipitor®.
10. Ibuprofen; p-isobutylhydratropic acid; advil®.
11. Naproxen; naprosyn®; anaprox®; bonyl®.
12. Tamoxifen.
13. Aminoglutethimide.
14. Hydrochlorothiazide; triamterene.
15. Acetylsalicylic acid; aspirin.
16. Cyclosporine; ciclosporin; neoral®.
17. Diclofenac; voltaren®; cataflam®.
18. Flecainide.
19. Propranolol; propranalol; propanolol; propanalol.
20. Verapamil.
21. Olanzapine.
22. Galanthamine; galantamine.

Category: gene

1. *Dopamine receptor d2*; *d2 dopamine receptor*; *dopamine d2 receptor.*
2. *Androgen receptor*; *dihydrotestosterone receptor*; *testosterone receptor*; *kennedy disease.*
3. *Hydroxymethylglutaryl-coa reductase.*
4. *Apolipoprotein e.*
5. *Leptin.*

6. *Peptidylprolyl isomerase a.*
7. *Apolipoprotein a-1*; *apolipoprotein a1*; *apolipoprotein a 1*; *apolipoprotein a-i*; *apolipoprotein ai*; *apolipoprotein a i.*
8. *Interleukin 2*; *t-cell growth factor*; *aldesleukin.*
9. *Plasminogen activator*; *urokinase.*
10. *Tumor necrosis factor precursor.*
11. *Tumor protein p53*; *tumor suppressor p53*; *phosphoprotein p53*; *tumor suppressor is p53.*
12. *Major histocompatibility complex class i*; *major histocompatibility complex class 1.*
13. *Major histocompatibility complex class ii.*
14. *Transforming growth factor*; *beta 1.*
15. *cgmp-binding cgmp-specific phosphodiesterase.*
16. *Von willebrand factor*; *von willebrand's factor*; *factor von willebrand*; *von willebrands factor.*
17. *Endothelin 1.*
18. *Hemoglobin alpha 2*; *hemoglobin alpha chain*; *alpha-globin.*
19. *Peroxisome proliferator-activated receptor gamma*; *ppar-gamma.*
20. *Beta-1 adrenergic receptor*; *beta-1 adrenoceptor.*

Category: target

1. Phosphodiesterase 5; cgmp specific phosphodiesterase type 5; 3',5'-cyclic-nucleotide phosphodiesterase; cyclic amp phosphodiesterase.
2. Cyclic-gmp phosphodiesterase.
3. Cyclooxygenase 1.
4. Cyclooxygenase 2; prostaglandin-endoperoxide synthase 2.
5. Estrogen receptor beta; er-beta.
6. Collagen receptor.
7. Glycoprotein iib/iiia receptor; gpiib-iiia receptor; glycoprotein iib/iiia; gp iib/iiia; gpiib/iiia receptor; fibrinogen receptor; platelet gpiib-iiia; platelet gpiib/iiia receptor; platelet glycoprotein iib-iiia.
8. Estrogen receptor alpha; er-alpha; oestrogen receptor.
9. 3-hydroxy-3-methylglutaryl coenzyme a reductase; hmg coa reductase; hmg-coa reductase.
10. c-reactive protein.
11. Progesterone receptor.
12. Epidermal growth factor receptor 2.
13. p2y purinoceptor; adp receptor; p2y receptor.
14. Serotonin receptor.
15. Cyclophilin a; peptidyl-prolyl cis-trans isomerase a; rotamase.
16. Acetylcholinesterase; cholinesterase.
17. Intrinsic factor.
18. Choline acetyltransferase; choline o-acetyltransferase; choline acetylase.
19. Acetylcholine.
20. Tryptophan hydroxylase.
21. Epinephrine; adrenaline.
22. Tumor necrosis factor-alpha; cachectin.
23. Tumor necrosis factor-beta; lymphotoxin.
